# Autologous Platelet Rich Fibrin Versus Steroid in Ultrasound-Guided Sacroiliac Joint Injection for Joint Dysfunction: Randomized Comparative Study

**DOI:** 10.5812/aapm-158219

**Published:** 2025-02-16

**Authors:** Omar Sayed Fargaly, Mohamed Ahmed Hamed, Maged Labib Boules, Mohammed Awad Alsaied, Mohammed Magdy Basiony, Mohammad Omar Mostafa, Amr Hamdy Mahmoud, Mohamed Ahmed Shawky

**Affiliations:** 1 Anesthesia Department, Faculty of Medicine, Fayoum University, Fayoum, Egypt; 2 Anesthesia Department, Fayoum University Hospital, Faculty of Medicine, Fayoum University, Fayoum, Egypt

**Keywords:** Platelet Rich Fibrin, Steroid, Ultrasound-Guided, Sacroiliac Joint Injection, Joint Dysfunction, Pain

## Abstract

**Background:**

Sacroiliac joint (SIJ) discomfort is frequently treated with steroids, although the relief is often temporary. The use of platelet-rich fibrin (PRF) may aid in tissue healing and prolong pain relief.

**Objectives:**

This study aims to compare the analgesic effects of a single autologous PRF injection with the commonly used steroid in ultrasound-guided intra-articular SIJ injections.

**Methods:**

This randomized clinical trial was conducted in the Department of Anesthesiology and Pain Management at Fayoum University Hospitals. The study included 94 adult patients with SIJ dysfunction confirmed by positive diagnostic tests. All patients were randomly assigned into two equal groups to receive either an ultrasound-guided PRF injection (group P, n = 47) or a steroid injection (group S, n = 47).

**Results:**

The Visual Analog Scale (VAS) score immediately after injection in group P was 7.51 ± 0.78 (mean ± SD), while in sroup S, it was 5.91 ± 0.54, with a highly significant difference between the two groups (P < 0.001). At six months, the VAS score was 3.51 ± 0.78 in group P compared to 4.68 ± 0.63 in group S, again showing a highly significant difference (P < 0.001). There was no significant difference in the Oswestry Disability Index (ODI) between the groups at one and six months.

**Conclusions:**

Intra-articular PRF injection is an effective therapy option for SIJ-related low back pain, offering prolonged pain relief compared to steroid injections.

## 1. Background

Approximately 20% of people suffer from low back pain, making it one of the most common ailments (1). In roughly 10 - 27% of these cases, the primary cause of discomfort has been identified as the sacroiliac joint (SIJ) (2).

Sacroiliac joint pain shares numerous symptoms with facet joint arthropathy and discogenic pain, making a definitive diagnosis challenging (3). Diagnosis can be established through a combination of medical history, clinical examination, imaging techniques, and diagnostic local anesthetic injections (4).

Injections of local anesthetics offer both therapeutic and diagnostic benefits and can be administered using various techniques, including landmark guidance (5), imaging assistance (6-8), or ultrasound guidance (9, 10). Compared to other guided injection modalities, ultrasound-guided injections offer several advantages, such as lower cost, real-time visualization of the needle, and reduced exposure to ionizing radiation (10).

Treatment modalities for SIJ arthropathy pain include physiotherapy (11), systemic analgesics such as non-steroidal anti-inflammatory drugs (NSAIDs), minimally invasive intra-articular and periarticular injections (12), radiofrequency neurotomy (13), and surgical fusion of the joint (14).

Various injectables have been used for intra-articular injections, most commonly local anesthetics and steroids (15). Recently, several biological growth factors present in human blood, particularly in platelets, have been injected intra-articularly to promote joint repair and provide longer-lasting pain relief by directly modifying the disease process. Platelet-rich plasma (PRP) has been utilized to deliver a high concentration of growth factors directly into the joint (16).

Platelet-rich fibrin (PRF), the second generation of PRP, is currently under investigation and offers advantages such as higher growth factor levels and simpler processing (17).

## 2. Methods

This research was conducted on 94 adult patients with SIJ dysfunction after obtaining written informed consent from each patient and receiving approval from the Fayoum University Ethics Committee and Institutional Review Board. The study was carried out from June 2021 to July 2022.

### 2.1. The Inclusion Criteria

Patients were required to be at least 18 years old, have experienced low back pain for more than six months, and have shown no response to conservative therapy for at least three months. Additionally, eligibility criteria included a positive result in at least one of the three provocation tests for SIJ discomfort—Patrick's Test, Gaenslen's Test, and compression & distraction test—as well as a positive diagnostic injection, defined as a reduction of more than 50% in pain on the Visual Analog Scale (VAS) following a local anesthetic injection.

### 2.2. The Exclusion Criteria

Patients were excluded from the study if they met any of the following criteria: Hip joint disorders, symptoms of lumbar radiculopathy, bleeding disorders (including those on anticoagulant therapy), a positive Kemp Test result (sciatica pain provocation test), active infection at the injection site, mental illnesses that impaired the ability to complete study-related questionnaires, a Body Mass Index (BMI) of 40 kg/m² or higher, or the presence of severe respiratory or cardiac diseases.

### 2.3. Randomization and Blinding

A random sequence number generator (RSNG) was used to assign 94 patients randomly to receive either PRF (group P, n = 47) or steroids (group S, n = 47). The randomization sequence was concealed in sealed envelopes, which were opened only after patient enrollment. Participants received either PRF or steroids according to the assignment indicated in the envelope. Both the participants and the data collectors were blinded to the type of injectate administered (PRF or steroid). Participant blinding was maintained by collecting 10 mL of blood from all patients, either for routine laboratory testing or for PRF preparation.

### 2.4. Patient Preparation

In accordance with the local protocol developed to assess these cases, a detailed history was taken, and a physical examination was performed, including the assessment of blood pressure and chest condition. Provocation tests, including Gaenslen's Test, Patrick's Test, and the compression and distraction test, were conducted. Additionally, laboratory investigations were performed, including an electrocardiogram (ECG), coagulation profile, liver function tests, serum creatinine, electrolytes, random blood glucose, and a complete blood count.

The VAS for pain (0 - 10), where 0 indicates no pain and 10 represents the worst imaginable pain, along with the details of the procedures, were explained to the participants prior to the intervention. As premedication, each patient received 2 mg of midazolam intravenously (IV). Intravenous access was established using a 20-gauge cannula, followed by the application of monitors, including a pulse oximeter, electrocardiography, and non-invasive blood pressure monitoring.

We used the Logiq P7 ultrasound scanner (GE Healthcare, Sunhwan, South Korea) equipped with a low-frequency convex ultrasound probe (4C, 2 - 5 MHz) and a Stimuplex D echogenic needle (B Braun, Germany; 22-gauge, 50 mm) for administering the injection.

For the preparation of injectable platelet-rich fibrin (iPRF), 10 mL of blood was drawn from each patient in the operating room, divided into three plain (non-citrated) tubes, following the method described by Abd El Raouf et al. (18). The samples were centrifuged at a low speed of 600 rpm (44 g) for eight minutes, within a maximum of two and a half minutes after collection. Upon completion of centrifugation, the tubes were carefully opened to avoid remixing the contents. Using a 5-mL syringe, 2 mL of iPRF was aspirated from the upper orange layer of the tubes, leaving the remaining blood components below.

### 2.5. Ultrasound Guided Technique

According to the method described by Jee et al. (10), the patient was positioned prone on a cushion. After cleaning the skin with povidone-iodine and draping the area, the ultrasound probe was placed transversely across the fifth lumbar spinous process and gradually moved downward until the sacrum was identified. The probe was then slightly shifted laterally and moved inferiorly to locate the second posterior sacral foramina. The first posterior sacral foramina was identified by a break in the hyperechoic contour of the sacral wing.

To visualize the sacrum, SIJ, and ilium, the lateral portion of the probe was slightly tilted upward. Color Doppler imaging was used to ensure there was no vascularization at the intended joint injection site. If vascular structures were detected, the probe was adjusted cranially or caudally until the area was free of vascularization.

After local anesthesia of the skin with 2 mL of 2% lidocaine, the injection was administered. For group P (PRF group), 3.5 mL of a combination of 2% lidocaine and PRF was injected into the SIJ. For group S (steroid group), 40 mg of methylprednisolone acetate (Depo-Medrol, Pfizer) (1 mL) combined with 2.5 mL of 2% lidocaine was administered into the joint.

### 2.6. Post-interventional Care

Prior to being transferred to the post-intervention care unit, patients were monitored for two hours following the procedure to assess heart rate, oxygen saturation, and any potential side effects.

All patients were prescribed the following medications: Diclofenac potassium 50 mg tablets three times daily (TID), diclofenac diethylamine gel applied topically three TID, and baclofen 10 mg tablets three TID.

### 2.7. The Primary Outcome

Pain assessment by VAS one month after injection.

### 2.8. The Secondary Outcomes

Pain was assessed using the VAS immediately after the procedure, as well as at one week, three months, and six months post-injection. The modified Oswestry Disability Index (ODI) was evaluated at one month and six months following the procedure. Patient satisfaction was measured on a scale from 0 to 3 (poor = 0, fair = 1, good = 2, excellent = 3). Additionally, demographic information, including age, weight, height, and BMI, was recorded, along with documentation of any complications or adverse events.

### 2.9. Statistical Analysis

The sample size was determined using G*Power 3.1.9.7, developed by the Department of Experimental Psychology at Heinrich Heine University, Germany. The calculation assumed an alpha error of 0.05 (two-tailed) and a beta error of 0.2, corresponding to a statistical power of 80%. Each group required 47 patients, with a clinical effect size of 0.28. A total of 104 participants were enrolled in the trial to account for an anticipated 10% dropout rate.

For statistical analysis, SPSS version 20 (Statistical Package for the Social Sciences, Chicago, IL, USA) was utilized to compare outcomes between the two groups. Categorical data were expressed as numbers and percentages, and the chi-square test was used for group comparisons. The Mann-Whitney U test was applied to assess differences between groups for nonparametric data, presented as medians and interquartile ranges. Parametric data were expressed as means ± standard deviations. A P-value of less than 0.05 was considered statistically significant, with a 95% confidence interval.

## 3. Results

The demographics of the study groups are presented in Table 1, showing no statistically significant differences (P = 0.95) between the two groups regarding age, sex, BMI, ASA grade, SIJ side, and weight.

**Table 1.** The Study Groups' Demographics and Side Distribution of Sacroiliac Joint Arthroplasty ^a, b^

Variables	Group P (n = 47)	Group S (n = 47)	Test of Significant	P-Value
**Gender**			χ^2^ = 0.044	0.834
Male	20 (42.55)	19 (40.43)		
Female	27 (57.45)	28 (59.57)		
**Age (y)**	49.21 ± 9.96	49.09 ± 9.79	*t* = 0.063	0.95
Range (min - max)	37 (32 - 69)	43 (29 - 72)		
**Height (cm)**	163.43 ± 3.02	166.21 ± 3.48	*t* = -4.145	< 0.001
**Weight (kg)**	92.72 ± 9.07	91.21 ± 8.67	*t* = 0.826	0.411
**BMI**	28.4 ± 3.57	27.89 ± 3.01	*t* = 0.756	0.452
**ASA grade**			χ^2^ = 0.103	0.748
I	42 (89.36)	41 (87.23)		
II	5 (10.64)	6 (12.77)		
**SIJ sidewise distribution**			χ^2^ = 0.05	0.823
Right	15 (31.91)	14 (29.79)		
Left	32 (68.09)	33 (70.21)		

Abbreviations: BMI, Body Mass Index; SIJ, sacroiliac joint.

^a^ Values are expressed as No. (%) or mean ± SD unless otherwise indicated.

^b^ χ^2^: Chi-square test; *t*: Independent *t*-test.

However, the height (cm) in group P had a mean ± SD of 163.4 ± 3, while in group S, the height (cm) had a mean ± SD of 166.2 ± 3.5. Although this difference holds little clinical significance, it was statistically significant (P < 0.001) between the two groups.

Table 2 shows no statistically significant difference in the VAS score before injection (P = 0.256) between the two groups. The VAS score significantly decreased in the steroid group immediately after injection, while the PRF group exhibited significantly lower VAS scores at 1 week, 1 month, 3 months, and 6 months (P < 0.001).

**Table 2.** Visual Analog Scale Score Among the Study Groups

Variables	Group P (n = 47)	Group S (n = 47)	*t*-Test	P-Value
**VAS score pre injection**			1.143	0.256
Mean ± SD	7.36 ± 1.17	7.09 ± 1.18		
Range (min - max)	4 (5 - 9)	5 (5 - 10)		
**VAS score immediately after injection**			11.531	< 0.001
Mean ± SD	7.51 ± 0.78	5.91 ± 0.54		
Range (min - max)	4 (6 - 10)	3 (4 - 7)		
**VAS Score at 1 week**			-5.646	< 0.001
Mean ± SD	3.04 ± 1.08	4.04 ± 0.55		
Range (min - max)	5 (1 - 6)	2 (3 - 5)		
**VAS Score at 1 month**			-10.293	< 0.001
Mean ± SD	2.47 ± 0.88	4.09 ± 0.62		
Range (min - max)	4 (1 - 5)	2 (3 - 5)		
**VAS Score at 3 months**			-18.392	< 0.001
Mean ± SD	1.98 ± 0.57	4.06 ± 0.53		
Range (min - max)	2 (1 - 3)	2 (3 - 5)		
**VAS Score at 6 months**			-8.026	<0.001
Mean ± SD	3.51 ± 0.78	4.68 ± 0.63		
Range (min - max)	4 (2 - 6)	4 (2 - 6)		

Abbreviation: VAS, Visual Analog Scale.

Table 3 demonstrates that the ODI score at 1 month in group P had a mean ± SD of 54.45 ± 17.9, while in group S, the ODI score at 1 month had a mean ± SD of 52.4 ± 19.35, with no statistically significant difference (P = 0.596) between the two groups. At 6 months, the ODI score in group P had a mean ± SD of 50.38 ± 16.43, compared to group S, which had a mean ± SD of 46.13 ± 16.21, again with no statistically significant difference (P = 0.209) between the two groups.

**Table 3.** Oswestry Disability Index Score Among the Study Groups ^a^

Variables	Group P (n = 47)	Group S (n = 47)	*t*-Test	P-Value
**ODI score at 1 month**	54.45 ± 17.9	52.4 ± 19.35	0.531	0.596
**ODI score at 6 months**	50.38 ± 16.43	46.13 ± 16.21	1.264	0.209

Abbreviation: ODI, Oswestry Disability Index.

^a^ Values are expressed as mean ± SD.

Table 4 indicates that immediate pain and stiffness showed a highly significant difference between the two study groups (P < 0.001). However, for complications such as chest pain, contralateral pain, giddiness, vasovagal attacks, and syncope, there were no statistically significant differences between the groups (P > 0.05). These assessments were conducted using a yes/no questionnaire based on complications reported in previous studies.

**Table 4.** Complications Incidence Among the Study Groups Immediate After Injection ^a^

Complications	Group P (n = 47)	Group S (n = 47)	Chi-square Test	P-Value
**Pain and stiffness**	21 (44.68)	2 (4.26)	20.78	< 0.001
**Epigastric pain**	2 (4.26)	2 (4.26)	0	1
**Giddiness**	2 (4.26)	1 (2.13)	0.344	0.557
**Contralateral pain**	2 (4.26)	1 (2.13)	0.344	0.557
**Vasovagal attack and syncope**	2 (4.26)	1 (2.13)	0	1

^a^ Values are expressed as No. (%).

## 4. Discussion

Numerous injectables, most frequently local anesthetics and steroids, have been employed for intra-articular injection; however, it has been found that they only provide temporary pain relief. We conducted this randomized clinical investigation at the Department of Anesthesiology, Fayoum University Hospitals, involving 94 adult individuals with SIJ dysfunction. All patients were randomly assigned into two equal groups to receive either a PRF injection (group P, n = 47) or a steroid injection (group S, n = 47).

Since there were no statistically significant differences between the study groups in terms of age, sex, weight, BMI, ASA classification, or SIJ side distribution, the current study included two well-matched groups with respect to all baseline data. This was done to exclude any potential confounding factors from the results. Although there was a statistically significant difference in height between the groups, this variation lacked clinical significance.

The current study revealed that both steroid and autologous PRF injections significantly decreased the VAS score, indicating pain reduction. However, at the one-week, one-month, three-month, and six-month post-intervention time points, the autologous PRF group showed significantly greater improvement. This may be attributed to the presence of various biological growth factors in human blood, particularly in platelets, which enhance tissue healing.

One of the most commonly used and reported questionnaires for assessing functional status in patients after spine surgery is the ODI. The ODI is a key metric utilized by many spine associations, as well as in routine medical practice, spine registries, and randomized clinical trials (19). It was found that there was no statistically significant difference between the PRF and steroid groups in terms of ODI at the 1- and 6-month follow-ups. This finding aligns with the results of Chen et al. (20), which also showed no significant difference in ODI at 6 months between the steroid and PRP groups. This may be due to the fact that the VAS score in our study was related only to static pain, while the ODI reflects dynamic pain associated with physical activity.

As stated by Hagg et al. (21), "If there was little change in other less important areas, the total score may lessen a notable improvement in the presenting problem. The capacity to sit, stand, and lift seems to improve less following therapy for persistent low back pain than sleep disruption, psychological irritation, and ability to do daily tasks. According to the study's findings, the back pain VAS is sensitive enough to identify the few clinically significant changes."

Our findings indicated that, while PRF appears to be the preferred long-term treatment, steroid injections offer greater immediate short-term outcomes.

The present study's findings were corroborated by Soliman et al. (22), who examined the efficacy of PRP and steroids in the treatment of SIJ pain through ultrasound-guided injections. The study included 35 patients in each group with comparable baseline data. Both steroid and PRP injections significantly decreased VAS and ODI scores immediately after treatment. However, after four weeks, PRP treatments demonstrated significantly lower VAS and ODI scores compared to steroid injections, indicating superior long-term benefits.

Our findings were also supported by Mohi Eldin et al. (23), who reported that PRF outperformed PRP, as patients receiving SIJ PRF injections showed notable clinical improvement at late follow-up compared to PRP recipients. Mohi Eldin et al. (23) analyzed two groups of SIJ injection recipients—124 PRF patients and 62 PRP patients—to evaluate pain reduction. The PRF group’s mean VAS score was 8.28 before injection, 5.06 one month later, and 4.61 six months later. In contrast, the PRP group’s mean pre-injection VAS was 8.29, with post-injection scores of 5.47 at one month and 5.19 at six months. The difference in VAS scores at six months was statistically significant (P = 0.045). However, comparisons of pre-injection and immediate post-injection VAS scores within each group (PRF or PRP) did not show significant differences (P = 0.909 and P = 0.154, respectively).

These findings suggest that while both PRF and PRP are effective in reducing SIJ-related pain, PRF may offer more sustained pain relief over time.

According to Singla et al. (16), which aligns with the findings of the current study, both the Modified Oswestry Disability Questionnaire (MODI) and VAS scores in the steroid group showed improvement for up to four weeks, followed by a decline at three months. In contrast, the PRP group demonstrated progressive improvement in both scores for up to three months, indicating more sustained benefits.

Ko et al. (24) reported that one patient who received a PRP injection into the SIJ ligaments experienced discomfort following the procedure. This discomfort may have been caused by the physiological effects of platelets or calcium, which can enhance the body’s natural inflammatory response, rather than being attributed to the injection technique itself.

Furthermore, a systematic review conducted by Ling et al. (25), which included five comparative studies, revealed no serious complications in any of the investigations. Minor complications were observed in three studies involving 64 PRP patients and 78 corticosteroid patients. The overall complication rates were moderate, with 12.5% in PRP patients and 12.8% in corticosteroid patients (P = 0.952). These minor complications included itching, hyperglycemia, and post-injection pain.

### 4.1. Conclusions

The findings of this study demonstrated that while PRF was more effective than steroids in reducing pain, steroid injections provided notable short-term improvements that gradually diminished over time. A greater number of patients in the PRF group reported significant pain relief. Given the absence of significant side effects, it can be concluded that PRF injection into the SIJ is a safe and effective treatment for low back pain caused by SIJ arthropathy.

## Data Availability

The dataset presented in the study is available on request from the corresponding author during submission or after publication.

## Clinical Trial Registration Code:

NCT04757740.
